# COVID‐19 and risk of retraumatization in adults with a past exposure to potentially traumatic events: A cross‐cultural exploration across Egypt, Germany, and Italy

**DOI:** 10.1002/jcop.22815

**Published:** 2022-02-10

**Authors:** Mariam Fishere, Eleonora Bartoli

**Affiliations:** ^1^ Department of Psychology Goethe University Frankfurt Germany

**Keywords:** adverse childhood experiences, COVID‐19, health locus of control, posttraumatic stress, traumatic experiences

## Abstract

**Aims:**

This study aims to: (1) explore the links between past exposure to potentially traumatic events, fear of contracting COVID‐19 and perceived stress; (2) investigate how the exposure to traumagenic experiences affects one's locus of control over their health; and (3) examine fear, stress reactions and differences in health locus of control across three different sociocultural contexts.

**Methods:**

A total of 524 adult participants were recruited from Egypt, Germany, and Italy through online channels. Self‐reporting instruments were used to assess previous exposure to potentially traumatic events, PTSD symptoms, fear of COVID‐19, perceived stress, and health locus of control.

**Results:**

Our findings highlight differences in reaction to COVID‐19 in relation to past exposure to potentially traumatic events and country of residence, both of which may inform tailored community‐based intervention practices.

**Conclusion:**

The impact of COVID‐19 might be particularly disruptive for people who survived potentially traumatic experiences. Nevertheless, the mass mental health impact of the COVID‐19 pandemic varies across different sociocultural contexts.

The coronavirus (COVID‐19) pandemic has introduced unique sources of traumatic stress to individuals the world over. At the time of writing, more than four million people have lost their lives due to COVID‐19 (WHO, 2021). Other factors, including the social and physical isolation imposed by lockdowns, financial challenges, and the apparent inability to control the virus from spreading, have had far‐ranging effects on our mental health. This has been particularly detrimental for individuals who have been previously exposed to other adversities or potentially traumatic experiences throughout their lives, and may already be living with a mental health disease (Campion et al., 2020; Yue et al., 2020). While similarities in responses to the pandemic can be observed between countries, COVID‐19 has laid bare the myriad fault lines in society, particularly inequality, as wide disparities in rates of infection and recovery according to place of residence (Kira et al., 2021) continue to be reported. Research into a wide range of traumatic experiences has routinely demonstrated a greater health burden on individuals who live in disadvantaged social and geographic areas. This study highlights the social inequality pertaining to the occurrence of potentially traumatic events by comparing adults' psychological well‐being during the early days of the pandemic across three countries: Egypt, Italy, and Germany. As the pattern of the COVID‐19 pandemic unraveled differently across the world, its toll on individuals' psychological health varied, depending on governmental policies, social norms, and community resilience. To better tackle the psychological impact of the pandemics, it is important to investigate the specific characteristics of each country by observing social norms and the prevalence of mental health issues preceding the outbreak of COVID‐19. For this reason, this study explores how Egypt, Germany, and Italy differ in changes in perceived stress and health locus of control in relation to the COVID‐19 outbreak.

## 1 THE PROCESSING OF POTENTIALLY TRAUMATIC EVENTS

Exposure to traumatic events is not uncommon. Traumatic events, whether natural or manmade, may trigger posttraumatic stress disorder (PTSD) and various psychopathological disorders. The ability to process a potentially traumatic event and the psychological impact experienced as a result depend on a number of characteristics related to the event, the individual and the community in which the event occurs (Knipscheer et al., 2020). The extent to which an event may cause long‐term negative effects is tied to: type (Baker et al., 2021), frequency (Yehuda & LeDoux, 2007), and age of onset (Zlotnick et al., 2008).

In terms of type, one main distinction can be done between manmade and natural traumas (Baker et al., 2021); while the former refers to experiences that entail a betrayal of trust in a relationship (e.g., childhood adversity and maltreatment, sexual assault, domestic violence), the latter includes life‐threatening experiences (e.g., earthquakes, car accidents, fire) that do not involve any interpersonal relationships (Briggs‐Gowan et al., 2010). In terms of frequency, revictimization—or being exposed to repeated traumas—increases the likelihood of developing trauma‐related symptoms (Suliman et al., 2009). That is because revictimization may activate the circuitries of fear and stress, and the severity and numerosity of such experiences (Yehuda & LeDoux, 2007).

The developmental repercussions incurred by exposure to trauma differ according to age of onset (Zlotnick et al., 2008). Adverse childhood experiences (ACEs), for example, comprise a range of potentially traumatic events, including child maltreatment, whether it be emotional, physical and sexual abuse, or emotional and physical neglect. When responsible adults abuse a relationship of trust and asymmetric power, which represents a wide range of interpersonal traumas, ACEs can potentially damage children's attachment patterns and the development of their emotional, cognitive and social skills, which may have long‐term psychopathological impact (Felitti et al., 1998).

With regard to the individual, multiple theories as well as empirical evidence highlight how childhood is a foundational period, during which individuals develop their sense of control over themselves and their environment (Ainsworth & Bowlby, 1991). ACEs may compromise children's development of a sense of control over their lives, laying the ground for a weak locus of control (Bryant & Trockel, 1976). This is large because when an adult perpetrator who is responsible for a child's safety fails to uphold that responsibility, the child develops ambiguous feelings towards them (Sousa et al., 2011). According to Janoff‐Bulman (1992), potentially traumatic experiences violate the sense of predictability and feelings of trust toward others as benevolent beings, particularly in the context of interpersonal relationships. Furthermore, when children live in environments that they are unable to manage, they develop an external locus of control, where the causality of events is attributed to either chance and other, external individuals (Roazzi et al., 2016). Analogously, traumatic events experienced during adulthood may be regarded as uncontrollable, or unavoidable, causing an individual to question their sense of agency (Mellon et al., 2009), including their own mental and physical health (Wielenga‐Boiten et al., 2015).

With regard to the community, a fair reading of the epidemiological literature would confirm that huge numbers of individuals around the world have suffered—or will at some point suffer from—traumatic experiences, including abuse, violence, and natural catastrophes (Knipscheer et al., 2020). However, these experiences are not randomly distributed (Roberts et al., 2011). Gender, socioeconomic class and sociocultural context have a great deal to do with who is at the greatest risk of developing different types of trauma.

Consequently, the heterogeneity of COVID‐19 experiences and responses across communities is irrefutable. The ecological perspective of community psychology offers a necessary look into the diverse sources and expressions of trauma‐related symptoms among trauma survivors. Individual differences in posttraumatic response and recovery are the result of complex interactions among individual and community factors. Therefore, investigations by community psychologists into the nature of wellness‐enhancing interventions and mechanisms of empowering social change can inform trauma‐focused interventions in different contexts (Harvey, 1996).

## 2 COVID‐19 AND PREDISPOSITIONS TO PTSD IN LOW‐ AND MIDDLE‐INCOME COUNTRIES

Current epidemiological data related to mental health problems are urgently required to generate accurate estimates of the burden of disease globally, and to inform an efficient and appropriate allocation of health resources, especially in low‐ and middle‐income countries. According to the world bank, low‐income countries, also known as developing countries, are those who have a per capita gross national income (GNI) of $1,025 or less. They usually face poor economic status, poor basic services, below‐average life expectancy, deteriorating infrastructure, and poor educational and health systems. As for middle‐income countries, they have a GNI that ranges between $1,025 and $12,475 and are considered to be important to the global economic growth. However, the global coverage of prevalence data for mental disorders in children and adolescents is still limited, with a significant underrepresentation of low‐ and middle‐income countries (Erskine et al., 2017), including Egypt. For PTSD, the literature indicates not only low availability of prevalence studies (Gunaratnam & Alisic, 2017), but also significant variability in PTSD rates in youth (Yatham et al., 2018). Besides, there could be cross‐cultural, regional, or other contextual differences reflecting significant variability in PTSD rates. Such variability could also be a result of methodological differences in sampling and measurement. Research shows that the rates and types of traumatic events to which individuals may be exposed to vary according to sociodemographic characteristics and country (Benjet et al., 2016), with many low‐ and middle‐income countries affected by armed conflict, violence and natural disasters, all of which have long‐term psychological impact (Suto, 2017). Consequently, the risk of onset and severity of PTSD may differ across cultural groups, which has led to the introduction of culture‐related diagnostic criteria for PTSD (DSM‐5). These diagnostic criteria were modified in the DSM‐5, which may not necessarily affect the overall PTSD prevalence rates, but may influence the prevalence of specific PTSD symptom criteria. These changes are especially important for low‐ and middle‐income countries, where additional epidemiological studies are needed.

The COVID‐19 pandemic has required numerous adjustments in almost everyone's daily life (Haleem et al., 2020). These adjustments perpetuate a sense of constant stress. Contagion‐related fear fosters a feeling of vulnerability due to the imminence, invisibility, and transmissibility of the virus (Pappas et al., 2009), which makes COVID‐19 appear threatening, uncontrollable, terrifying, and thus more stressful. While the pandemic can be described as a collective experience that is potentially traumatic, evidence shows that only a minority of people develop PTSD after exposure to a traumatic event, suggesting that individuals vary in predispositions (Voges & Romney, 2003). Therefore, when assessing the effects of the spread of COVID‐19, it is vital to understand such predispositions among individuals, including past exposure to adverse childhood experiences (ACEs) and other potentially traumatic experiences that unfolded across different developmental phases. Although the consequences of exposure to ACEs and other potentially traumatic events cannot be assessed in the short run, high levels of stress can increase a person's predisposition to develop a negative response to an additional, uncontrollable, and potentially traumatic event such as the spread of COVID‐19.

The concept of Health Locus of Control (Wallston et al., 1978) lists three ways by which one exhibits control over their own health: (1) “internal” health locus of control indicates a belief that one's health is controlled by one's own actions; (2) “powerful others” health locus of control reflects the expectation that others can influence the outcome of one's health; and (3) “chance” health locus of control refers to the belief that chance is the main determinant of one's health. Individuals who employ internal health locus of control are usually associated with higher levels of self‐reported physical well‐being; they tend to cope more successfully with health‐related problems. Studies have highlighted that being exposed to ACEs and potentially traumatic events influence the individual's locus of control. For example, Roazzi et al. (2016) found higher rates of externality (i.e., attribution of control to others and to chance) in children who had lived through ACEs than in children who had not. Similarly, Mellon et al. (2009) found higher rates of externality in adults living in areas that were often subjected to potentially traumatic events (e.g., wildfires) than in adults living in areas that were not exposed to such risks. Conversely, Mallary (2020) observed higher feelings of control over the course of illness in cancer patients who had gone through different potentially traumatic events, including ACEs.

## 3 THE PRESENT STUDY

The COVID‐19 global pandemic still represents a major global threat with detrimental health consequences. This study aims to further‐develop the understanding of the consequences of COVID‐19 within the framework of PTSD. Focusing on risk factors or pre‐existing conditions that may proliferate the negative consequences of the pandemic is crucial to provide interventions for those who might be at a higher risk of developing mental health conditions. Some individuals—as well as communities—are at a higher risk of exposure to trauma. This exploratory study focuses on Egypt, a low‐ and middle‐income country and one of the biggest Arab countries, for three main reasons: First, Egypt is undergoing a number of psychosocial changes associated with the sociopolitical turmoil that has been unfolding over the last few years, which increased community violence and, possibly, several other forms of violence (Matthies‐Boon, 2017); the literature on ACEs and potentially traumatic events in Egypt is scarce (Antai et al., 2016); and third, given the high rates of migration from other Arab countries to the West, it is crucial to have both insight into and evidence from the conceptualization of ACEs and potentially traumatic events in Egypt to develop culturally‐appropriate interventions for particularly vulnerable populations (Silove et al., 2017).

This study aims at investigating how past experiences with ACEs and potentially traumatic events affect individual responses to COVID‐19. Specifically, our first aim was to compare fear of contracting COVID‐19, perceived stress, and changes in health locus of control in participants who were only exposed to ACEs. We then examined the three variables by looking at three different groups: individuals who were only exposed to potentially traumatic events, individuals who were exposed to both ACEs and potentially traumatic events, and individuals who were not exposed to either, namely the control group. Our second aim was to investigate whether participants in any of the potentially traumatic events groups had different health locus of control than the control group. Lastly, Our third aim was to identify the models that better predicted developing a fear of contracting COVID‐19, levels perceived stress, and changes in health locus of control. Accordingly, we hypothesized the following:

- Fear of contracting COVID‐19 and levels of perceived stress are higher in the three potentially traumatic events groups than in the control group (H1).
- Participants with a history of ACEs, other potentially traumatic events or both will score higher on the “powerful others” health locus of control scale (H2).
- A positive association between exposure to ACEs and/or potentially traumatic events, PTSD, and all our outcome variables (H3).

## 4 METHOD

### 4.1 Participants

Participants were recruited online in June 2020. Flyers were designed in Arabic, English, German, and Italian. They were disseminated through social media channels, e‐newsletters, and word‐of‐mouth. Inclusion criteria were being at least 18 years of age and residing in one of the following countries during the COVID‐19 outbreak: Egypt, Germany, and Italy.

The final sample includes 524 adults (*M* = 35.97 years; *SD* = 11.86; 62% female; 58.2% residing in Egypt, 29.0% residing in Germany, and 12.8% residing in Italy). While it is noticeable that females are overrepresented in our sample, it is important to highlight that this is common in similar research (Dickinson et al., 2012). Moreover, statistical considerations have been taken to achieve gender balance in the sample. Most of the participants were in a relationship (58.8%, *N* = 308), 33.4% were single (*N* = 175), 5.5% divorced (*N* = 29), 0.6% widowed (*N* = 3), and 1.7% marked “other” under relationship status (*N* = 9). 62% of the participants (*N* = 327) had no children and the remaining part had at least one child (*M* = 0.74 children per participant; *SD* = 1.12).

The sample is mainly composed of participants with a higher education degree (84.7%, *N* = 443); 12.8% of participants completed the secondary degree of education (*N* = 67) and 2.5% primary school (*N* = 13). Concerning their employment, 65.6% of the participants were employed (11.7% of whom worked as health professionals, of which 4.2% in COVID‐19 Units). Concerning their employment at the time of the study, 65.6% of participants were employed (*N* = 343), 13.8% were students (*N* = 72), 17.6% were unemployed (*N* = 92) and 3.1% were retired (*N* = 16). Among the 343 employed participants, 17.8% (*N* = 61) were health professionals, 36.1% of whom (*N* = 22) worked with COVID‐19 patients.

Health‐related information was also collected, since some existing health conditions could increase the vulnerability to COVID‐19 in terms of both severity of symptoms following the contraction of the virus and impact on mental health. Most participants did not present with any chronic diseases at the time of the study (65.3%, *N* = 342) and did not consult any mental health services (79.2%, *N* = 415). The majority of the sample had not contracted COVID‐19 (90.8%, *N* = 476), (66.6%, *N* = 349) didn't know anyone who did, and (89.3%, *N* = 468) didn't lose any acquaintances due to the virus.

Based on the participants' exposure to potentially traumatic events, be they Adverse Childhood Experiences (ACEs) or other potentially traumatic events, four groups were created: (1) participants who did not report being exposed to any potentially traumatic event (11.8%), that is the control group; (2) participants who were exposed to ACEs but not to other potentially traumatic events (23.5%); (3) participants who were exposed to potentially traumatic events but not any ACEs (14.9%); (4) participants who were exposed to both ACEs and other potentially traumatic events (49.8%). Socio‐demographic characteristics and health‐related information of the sample and of each group are reported in the tables for descriptive purposes (see Tables 1 and 2).

**Table 1:** Socio‐demographic characteristics per group of exposure to adverse childhood experiences ACEs and/or other potentially traumatic experiences (PTE)

	Control *n* (%)	ACEs only *n* (%)	PTEs only *n* (%)	ACEs and PTEs *n* (%)	Overall *n* (%)
Age (*M, SD*)	33.94 (12.46)	33.85 (11.17)	38.55 (14.0)	36.67 (11.11)	35.97 (11.84)
Gender					
Female	37 (59.7)	84 (68.3)	49 (62.8)	156 (59.8)	326 (62.2)
Male	25 (40.3)	39 (31.7)	28 (35.9)	102 (39.1)	194 (37.0)
Other			1 (1.3)	3 (1.2)	4 (0.8)
Residence					
Egypt	16 (25.8)	67 (54.5)	41 (52.6)	181 (69.3)	305 (58.2)
Germany	35 (56.5)	38 (30.9)	25 (32.1)	52 (20.7)	152 (29.0)
Italy	11 (17.7)	18 (14.6)	12 (15.4)	26 (10.0)	67 (12.8)
Relationship status					
Single	86 (33.0)	14 (22.6)	52 (42.3)	23 (29.5)	175 (33.4)
In a relationship	153 (58.6)	43 (69.3)	63 (51.2)	49 (62.8)	308 (58.8)
Divorced	19 (7.3)	1 (1.6)	5 (4.1)	4 (5.1)	29 (5.5)
Widowed	3 (1.1)	4 (6.4)	1 (0.8)	2 (2.6)	3 (0.6)
Other			2 (1.6)		9 (1.7)
Children (*M, SD*)	0.55 (0.88)	0.59 (1.04)	0.88 (1.12)	0.82 (1.20)	0.74 (1.12)
Education					
Primary	1 (1.6)	1 (0.8)	3 (3.9)	8 (3.1)	13 (2.5)
Secondary	13 (21.0)	18 (14.6)	8 (10.4)	28 (10.7)	67 (12.8)
Undergraduate	23 (37.1)	60 (48.8)	30 (39.0)	118 (45.2)	231 (44.2)
Graduate	25 (40.3)	44 (35.8)	36 (46.8)	107 (41.0)	212 (40.5)
Employment					
Student	15 (24.2)	22 (17.9)	13 (16.9)	22 (8.4)	72 (13.8)
Employed	38 (61.3)	81 (65.9)	49 (63.6)	175 (67.0)	343 (65.6)
Unemployed	7 (11.3)	18 (14.6)	9 (11.7)	58 (22.2)	92 (17.6)
Retired	2 (3.2)	2 (1.6)	6 (7.8)	6 (2.3)	16 (3.1)
Health professional					
Yes	6 (9.7)	12 (9.8)	12 (15.6)	31 (11.9)	61 (11.7)
No	56 (90.3)	106 (86.2)	61 (79.2)	218 (83.5)	441 (84.3)
Other		5 (4.1)	4 (5.2)	12 (4.6)	21 (4.0)
Working with COVID‐19 patients					
Yes	3 (4.8)	2 (1.6)	3 (3.8)	14 (5.4)	22 (4.2)
No	59 (61.2)	120 (82.8)	74 (98.7)	247 (85.1)	522 (95.8)

**Table 2:** Health‐related information per group of exposure to adverse childhood experiences (ACEs) and/or other potentially traumatic experiences (PTE)

	Control *n* (%)	ACEs only *n* (%)	PTEs only *n* (%)	ACEs and PTEs *n* (%)	Overall *n* (%)
Chronic illnesses					
Yes	8 (12.9)	36 (29.3)	17 (21.8)	121 (46.4)	182 (34.7)
No	51 (82.3)	86 (69.9)	59 (75.6)	137 (52.5)	342 (65.3)
Other	3 (4.8)	1 (0.8)	2 (2.6)	3 (1.1)	
Current use of mental health services					
Yes	6 (9.7)	26 (21.1)	13 (16.7)	64 (24.5)	109 (20.8)
No	56 (90.3)	97 (78.9)	63 (83.3)	197 (75.5)	415 (79.2)
COVID‐19 diagnosis					
Yes	3 (4.8)	13 (10.6)	7 (9.0)	23 (8.8)	46 (8.8)
No	59 (95.2)	108 (87.8)	71 (91.0)	238 (91.2)	476 (90.8)
Other		2 (1.6)			2 (4.4)
COVID‐19 acquaintance diagnosis					
Yes	37 (59.7)	83 (67.5)	51 (65.4)	178 (68.2)	349 (66.6)
No	25 (40.3)	40 (32.5)	27 (34.6)	83 (31.8)	175 (33.4)
COVID‐19 acquaintance death	7 (11.3)	15 (12.2)	4 (5.1)	30 (11.5)	56 (10.7)
Yes	55 (88.7)	108 (87.8)	74 (94.9)	231 (88.5)	468 (89.3)
No					

### 4.2 Procedure

A cross‐sectional web‐based survey design was adopted. Ethical approval for this study was obtained from the Institutional Review Board at the Goethe University Frankfurt, Germany. Online consent was obtained from all participants, and they were allowed to terminate the survey at any time they desired. The survey was anonymous, and confidentiality of information was assured. The study was divided into five sections. The first section included sociodemographic and background information questions, which were designed for the present study. The second section inquired about health locus of control, fear of contracting COVID‐19, and perceived stress. The third section was centered on the assessment of past exposure to trauma, whether in childhood, adolescence, or adulthood. The fourth section assessed the participants' resilience and emotion regulation strategies. In the fifth and last section, participants were asked to reflect on and narrate their experiences with the pandemic. Data for the fourth and fifth sections will be reported elsewhere. All participants received contact information for free mental health services available in their country of residence.

### 4.3 Measures

#### 4.3.1 Adverse childhood experiences questionnaire

Participants were asked about their exposure to potentially traumatic events before the age of 18 using the Adverse Childhood Experiences Questionnaire (ACE‐Q; Felitti et al., 1998). The ACE‐Q includes 10 yes/no self‐report items assessing exposure to physical, emotional, and sexual abuse, household dysfunction, including witnessing domestic violence, substance abuse, mental illness, and incarceration. Higher scores indicate a higher number of potentially traumatic events in childhood. It has adequate internal consistency (*α* = 0.88) and construct validity (Zarse et al., 2019).

#### 4.3.2 Primary care posttraumatic stress disorder screen

Lifetime exposure to potentially traumatic experiences was assessed using the Primary Care Posttraumatic Stress Disorder Screen (PC‐PTSD‐5; Prins et al., 2016). The PC‐PTSD is a self‐report screen composed of five yes/no items with a score ranging from 0 to 5. Higher scores indicate higher exposure to potentially traumatic events.

#### 4.3.3 Posttraumatic stress disorder checklist for DSM–5

The current posttraumatic symptoms were investigated with the Posttraumatic Stress Disorder Checklist for DSM–5 (PCL‐5; Weathers et al., 2013). It includes 20 items on a 5‐point scale (ranging from “0 = Not at all” to “4 = Extremely”), assessing trauma‐related symptoms experienced in the past month. The total symptom severity score, ranging from 0 to 80, is obtained by summing the scores of each item. The score of 33 points is suggested to be the cutoff for a possible diagnosis of PTSD. The PCL‐5 has strong internal consistency (*α* = 0.94), and convergent validity (*r* = 0.74–0.85) (Blevins et al., 2015). The PCL‐5 was answered based on participant's choice of their most traumatic experience, whether it had taken place during childhood or adulthood.

#### 4.3.4 Fear of contracting COVID‐19

The Fear of COVID‐19 (Ahorsu et al., 2020) is a seven‐item measure, with good internal consistency (*α* = 0.82), assessing the individual's fear of COVID‐19 on a 5‐point Likert scale (ranging from totally disagree to totally agree). The total score ranges from 7 to 35 and is obtained by summing up each item score. The higher the score, the greater the fear of COVID‐19.

#### 4.3.5 Perceived stress scale

Participants were asked to rate the extent to which they experienced negative emotions they identified as stress over the past month with the four‐item Perceived Stress Scale (PSS‐4; Cohen, 1983). The PSS‐4 assesses respondents' perceived stress on a 5‐point scale. The total score is obtained by reversing the scores of the two positively phrased items and then summing the scores of each item. Higher scores indicate higher stress. The scale has acceptable internal consistency (α = 0.77; Warttig et al., 2013).

#### 4.3.6 Multidimensional health locus of control

The Multidimensional Health Locus of Control (MHLC; Wallston et al., 1978) includes 18 items assessing respondents' belief of control and causal attributions over their health on a 5‐point scale. The questionnaire is composed of three subscales, each composed of six items, assessing internality (i.e., the belief the person has control over their own health), chance externality (i.e., the belief that health is a matter of destiny and chance), and powerful others externality (i.e., the belief that other participants have power and control over one's own health.) Scores are calculated separately for each subscale and higher scores indicate higher degrees of the specific type of health locus of control that the subscale measures.

### 4.4 Data analysis plan

Assumptions of multicollinearity, linearity, homoscedasticity, and normal distribution were met. Sixteen outliers were winsorized. While it is noticeable that females are overrepresented in our sample, it is important to highlight that this is common in similar research (Dickinson et al., 2012). Statistical considerations have been taken to achieve gender balance in the sample. General linear model procedures (GLM) and multiple analyses of variance (MANOVA) were used to test the hypotheses concerning group differences. The Tukey post‐hoc tests were used to follow up on significant results. Multiple regressions were run to test the hypotheses concerning relationships between continuous variables.

## 5 RESULTS

### 5.1 Descriptive statistics

Study descriptive statistics and correlations between study variables are reported for descriptive purposes (see Tables 3 and 4).

**Table 3:** Descriptive statistics of ACEs and/or other PTEs, PTSD symptoms, fear of COVID‐19, perceived stress, and HLC per group of exposure to ACEs and/or other PTEs and in the whole sample

	Control *n* (%)	ACEs only *n* (%)	PTEs only *n* (%)	ACEs and PTEs *n* (%)	Overall *n* (%)
Number of ACEs (*M; SD*)		2.33 (1.38)		3.03 (1.68)	2.05 (1.86)
Number of PTEs (*M; SD*)			1.60 (0.76)	2.03 (1.10)	1.25 (1.25)
Number of ACEs and PTEs (*M; SD*)		2.33 (1.38)	1.60 (0.76)	5.06 (2.24)	3.31 (2.55)
PTSD (*M; SD*)	33.10 (13.59)	45.28 (17.97)	37.03 (13.99)	48.45 (18.50)	44.19 (18.10)
Fear of COVID‐19 (*M; SD*)	14.21 (5.28)	16.20 (5.20)	16.44 (5.03)	16.62 (5.86)	16.21 (5.56)
Perceived stress (*M; SD*)	10.42 (3.13)	12.27 (3.19)	10.86 (2.86)	12.39 (3.03)	11.90 (3.14)
HLC internal (*M; SD*)	19.95 (3.22)	20.84 (3.48)	20.09 (2.87)	20.44 (3.21)	20.42 (3.23)
HLC chance (*M; SD*)	16.31 (3.97)	16.01 (4.06)	15.77 (3.45)	16.74 (3.89)	16.37 (3.89)
HLC others (*M; SD*)	15.35 (4.13)	17.11 (3.84)	17.51 (3.07)	17.06 (3.29)	16.94 (3.55)

*Note*: *N* = 524.

Abbreviations: ACE, adverse childhood experience; PTE, potentially traumatic event; PTSD, posttraumatic stress disorder; HLC, health locus of control; COVID‐19, novel coronavirus‐19.

**Table 4:** Correlations for study variables

	1	2	3	4	5	6	7	8	9
1. ACE	—								
2. PTE	0.32**	—							
3. ACE and PTE	0.88**	0.72**	—						
4. PTSD	0.41**	0.24**	0.41**	—					
5. Fear of COVID‐19	0.15**	0.15*	0.18**	0.36**	—				
6. Perceived Stress	0.28**	0.08	0.25**	0.59**	0.35**	—			
7. HLC Internal	0.02	−0.02	0.01	−0.03	0.01	−0.13**	—		
8. HLC Chance	0.12**	0.08	0.13**	0.15**	0.16**	0.17**	−0.14**	—	
9. HLC Others	0.10*	0.09*	0.12**	−0.15**	0.29**	0.14**	0.25**	0.10*	—

*Note*: All variables are continuous.

Abbreviations: ACE, adverse childhood experience; PTE, potentially traumatic event; PTSD, posttraumatic stress disorder; HLC, health locus of control; COVID‐19, novel coronavirus‐19.

** *p* < 0.001

* *p* < 0.05.

Hypothesis 1 (H1), which states that participants who were exposed to adverse childhood experiences (ACEs) and other potentially traumatic events perceive more COVID‐19‐related stress than the other three groups (namely, those who have been exposed to ACEs only, those who were exposed to other potentially traumatic events, and those who have not been exposed to either) was assessed using a MANOVA. H1 was partially supported. Results showed a significant difference in means of fear of contracting COVID‐19 (*F*[3, 520] = 3.223; *p* < 0.05) and perceived stress (*F*[3, 520] = 10.651; *p* < 0.001) between groups. The Tukey HSD post‐hoc test showed that the group who had experienced both ACEs and potentially traumatic events (*M* = 16.62; *SD* = 5.86) had significantly higher scores on fear of COVID‐19 scale than the control group (*M* = 14.21; *SD* = 5.28). This was not the case when compared to the two other groups. The group with both ACEs and potentially traumatic events scored significantly higher on the perceived stress scale (*M* = 12.39; *SD* = 3.032) than all three groups: the control group (*M* = 10.42; *SD* = 3.139), the history of ACEs only group (*M* = 12.27; *SD* = 3.193), and the group that was exposed to other potentially traumatic events and not ACEs (*M* = 10.86; *SD* = 2.864).

Hypothesis 2 (H2), which foreshadows that participants with adverse experiences score higher on the powerful others health locus of control than participants in the control group, was also tested using a MANOVA. The hypothesis was supported (*F*[3, 520] = 5.122; *p* = 0.002). The Tukey HSD post‐hoc test showed that the ACEs only group (*M* = 17.11; *SD* = 3.84), the potentially traumatic events group (*M* = 17.51; *SD* = 3.07), and the group with both ACEs and other potentially traumatic events (*M* = 17.06; *SD* = 3.29) had significantly higher scores on powerful others health locus of control than the control group (*M* = 15.35; *SD* = 4.13).

Hypothesis 3 (H3), which states that exposure to ACEs or other potentially traumatic events, as well as PTSD symptoms, predict COVID‐19‐related stress and a more external health locus of control, was tested using multiple regressions. The hypothesis was supported. Exposure to ACEs or potentially traumatic events and PTSD symptoms predicted fear of contracting COVID‐19, higher rates of perceived stress, powerful others health locus of control, and chance locus of control (*R* ^2^ = 0.219; *p* < 0.001) (see Table 3). However, it did not predict internal health locus of control (Table 5).

**Table 5:** Multiple regression with covariates and ACEs, PTEs, and PTSD predicting fear of COVID‐19, perceived stress, chance, and powerful others health locus of control

	Predictors	*B*	*SE*	95% CI
Fear of COVID‐19	Constant	8.74**	2.06	(4.67, 12.76)
	ACEs	−0.06	0.13	(−0.34, 0.21)
	PTEs	0.17	0.21	(−0.25, 0.56)
	PTSD	0.11**	0.02	(0.08, 0.14)
	Age	0.02	0.02	(−0.02, 0.06)
	Gender	−1.85**	0.45	(−2.73, −1.07)
	Relationship	−0.13	0.50	(−1.12, 0.72)
	Children	0.26	0.26	(−0.27, 0.80)
	Education	2.49**	0.68	(0.80, 3.78)
	Employment	−0.18	0.46	(−1.05, 0.63)
	Health professional	1.63*	0.78	(0.07, 3.33)
	COVID‐19 patients	−0.29	1.35	(−2.75, 1.95)
	Chronic illness	−0.58	0.52	(−1.76, 0.52)
	Health services	0.16	0.61	(−1.01, 1.36)
	COVID‐19 diagnosis	0.07	0.90	(−1.85, 2.08)
	COVID‐19 acquaintance	−1.07*	0.49	(−1.97, −0.18)
	COVID‐19 death	−0.23	0.74	(−1.69, 1.29)
Perceived stress	Constant	8.95**	1.09	(6.89, 11.22)
	ACEs	0.11	0.07	(−0.03, 0.24)
	PTEs	−0.15	0.09	(−0.34, 0.02)
	PTSD	0.09**	0.01	(0.07, 0.10)
	Age	−0.03*	0.01	(−0.05, −0.01)
	Gender	0.33	0.24	(−0.16, 0.79)
	Relationship	−0.25	0.24	(−0.70, 0.20)
	Children	0.02	0.13	(−0.23, 0.28)
	Education	0.25	0.36	(−0.47, 0.93)
	Employment	0.50*	0.25	(0.00, 0.99)
	Health professional	0.12	0.39	(−0.68, 0.91)
	COVID‐19 patients	0.03	0.62	(−1.10, 1.19)
	Chronic illness	−0.57*	0.26	(−1.07, −0.12)
	Health services	0.10	0.30	(−0.47, 0.72)
	COVID‐19 diagnosis	−0.43	0.38	(−1.21, 0.39)
	COVID‐19 acquaintance	−0.38	0.26	(−0.89, 0.09)
	COVID‐19 death	0.44	0.33	(−0.15, 1.05)
HLC Chance	Constant	15.44	1.51	(12.31, 18.18)
	ACEs	0.11	0.10	(−0.10, 0.30)
	PTEs	−0.03	0.15	(−0.36, 0.27)
	PTSD	0.02*	0.012	(0.00, 0.05)
	Age	−0.02	0.02	(−0.05, 0.01)
	Gender	0.79*	0.35	(0.09, 1.59)
	Relationship	−0.67	0.39	(−1.42, 0.12)
	Children	0.76**	0.18	(0.40, 1.09)
	Education	−0.44	0.57	(−1.55, 0.69)
	Employment	0.41	0.37	(−0.35, 1.13)
	Health professional	0.97	0.68	(−0.35, 2.1)
	COVID‐19 patients	−1.68	0.10	(−3.89, 0.49)
	Chronic illness	0.23	0.38	(−0.50, 0.94)
	Health services	0.12	0.46	(−0.81, 1.01)
	COVID‐19 diagnosis	−0.04	0.53	(−1.07, 1.01)
	COVID‐19 acquaintance	−0.17	0.38	(−0.88, 0.43)
	COVID‐19 death	0.71	0.59	[−0.41, 1.91]
HLC Powerful others	Constant	15.39**	1.44	(12.40, 18.46)
	ACEs	0.05	0.08	(−0.11, 0.22)
	PTEs	0.02	0.13	(−0.24, 0.30)
	PTSD	0.02*	0.01	(0.00, 0.04)
	Age	−0.01	0.02	(−0.04, 0.026)
	Gender	0.40	0.30	(−0.19, 0.99)
	Relationship	−1.10*	0.37	(−1.80, −0.40)
	Children	0.55*	0.17	(0.22, 0.92)
	Education	1.47*	0.49	(0.46, 2.44)
	Employment	0.31	0.33	(−0.29, 0.92)
	Health professional	0.23	0.57	(−1.02, 1.51)
	COVID‐19 patients	−0.36	1.07	(−2.57, 1.57)
	Chronic illness	−0.28	0.34	(−0.92, 0.39)
	Health services	0.94*	0.38	(0.23, 1.67)
	COVID‐19 diagnosis	−0.43	0.52	(−1.36, 0.55)
	COVID‐19 acquaintance	−0.19	0.35	(−0.90, 0.52)
	COVID‐19 death	−0.61	0.47	(−1.50, 0.22)

*Note*: ACEs indicate total number of ACEs. PTEs indicate total number of potentially traumatic experiences after childhood and adolescence. PTSD = PCL‐5 total score. Gender: 0 = female, 1 = male. Relationship: 0 = single, 1 = in a relationship. Education: 0 = high school degree, 1 = graduate or undergraduate degrees. Employment: 0 = unemployed, 1 = employed. Health professional: 0 = yes, 1 = no. COVID‐19 patients: 0 = working with COVID‐19 patients, 1 = not working with COVID‐19 patients. Chronic illness: 1 = yes, 0 = no. Health services: 0 = yes, 1 = no. COVID‐19 diagnosis: 0 = yes, 1 = no. COVID‐19 acquaintance: 0 = an acquaintance was diagnosed with COVID‐19, 1 = not an acquaintance was diagnosed with COVID‐19. COVID‐19 death: 0 = know someone who died due to COVID‐19, 1 = does not know someone who died due to COVID‐19. b = Unstandardized coefficient; SE = Standard error. The confidence interval for the coefficient b is a BCa Bootstrapped CI based on 1000 samples.

** *p* < 0.001

* *p* < 0.05.

### 5.2 Exploratory analyses

**Figure 1:**
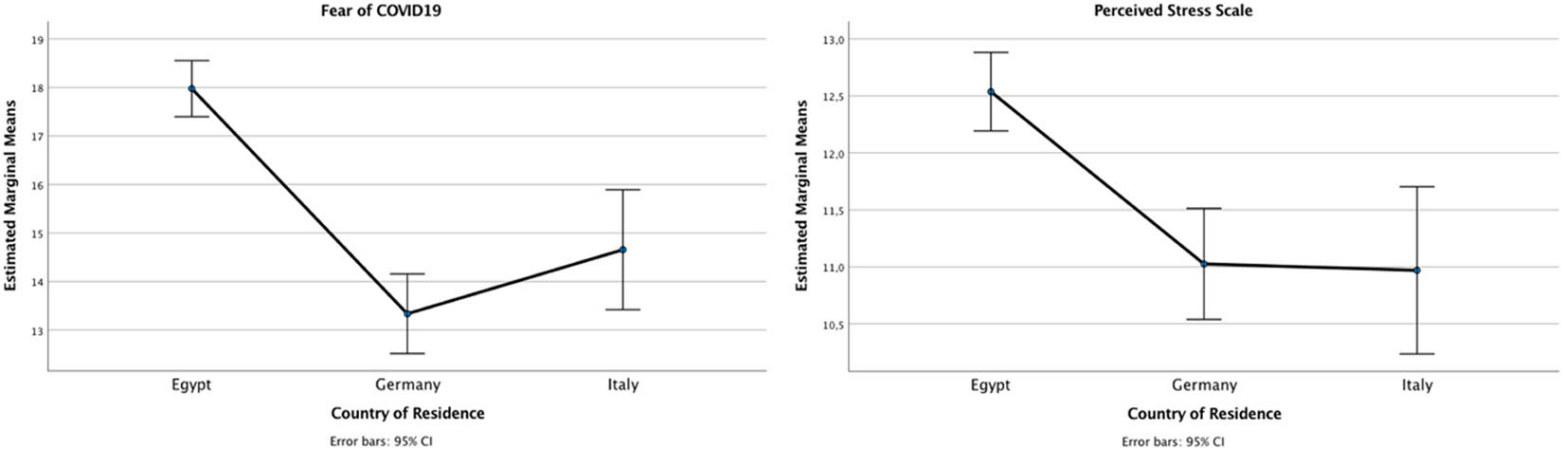
Mean scores and standard deviations per country in Fear of Covid‐19 and Perceived Stress

We were interested in exploring potential cross‐cultural differences by looking at fear of contracting COVID‐19, levels of perceived stress, and changes in health locus of control across adults residing in Egypt, Germany, and Italy. A MANOVA highlighted a significant difference in means of all outcome variables, namely fear of COVID‐19 (*F*[2, 521] = 44.724; *p* < 0.001), perceived stress (*F*[2, 521] = 15.947; *p* < 0.001), internal health locus of control (*F*[2, 521] = 8.190; *p* < 0.001), chance health locus of control (*F*[2, 521] = 23.792; *p* < 0.001), and powerful others health locus of control (*F*[2, 521] = 60.886; *p* < 0.001). The Tukey HSD post‐hoc test showed that participants in Egypt reported higher levels of fear of contracting COVID‐19 (*M* = 17.98; *SD* = 5.467), as compared to participants in Germany (*M* = 13.34; *SD* = 4.850) or Italy (*M* = 14.66, *SD* = 4.202). Also, Egyptian participants perceived higher levels of stress (*M* = 12.54; *SD* = 2.975) than their German (*M* = 11.03; *SD* = 3.285) and Italian counterparts (*M* = 10.97, *SD* = 2.881). Figure 1 provides a visual representation of these results.

**Figure 2:**
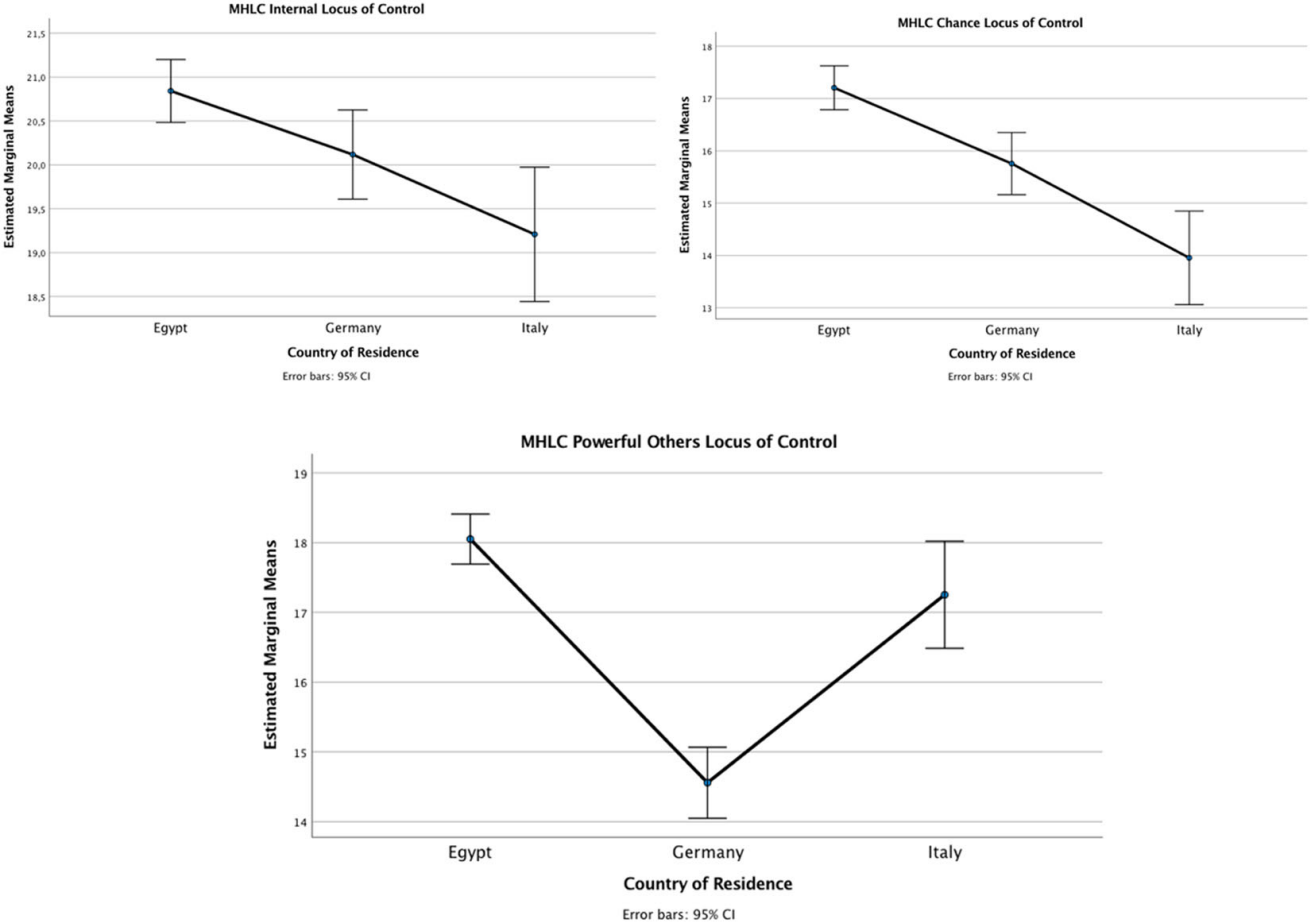
Mean scores and standard deviations per country in Internal Health Locus of Control, Chance Health Locus of Control and Powerful Others Health Locus of Control

Concerning the internal health locus of control, scores were found to be significantly higher in Egypt (*M* = 20.84, *SD* = 3.249) than in Italy (*M* = 20.12; *SD* = 3.239). No significant differences were found between Germany and the two other countries studied. In terms of externality of the health locus of control, participants in Egypt scored higher in chance health locus of control (*M* = 17.21; *SD* = 3.453) when compared to participants in Italy (*M* = 13.96, *SD* = 4.304) and Germany (*M* = 15.76; *SD* = 3.982), which came at the top. With regard to attribution of control over health to powerful others, participants in Germany obtained significantly lower scores in powerful others health locus of control (*M* = 14.56; *SD* = 3.673) than participants in both Egypt (*M* = 18.05; *SD* = 2.991) and Italy (*M* = 17.25; *SD* = 2.946) (Figure 2).

## 6 DISCUSSION

The outbreak of COVID‐19 presents new challenges for mental health, especially for individuals who have a history of exposure to potentially traumatic events during childhood, early adulthood, or both. In this study, we report on the implications of COVID‐19 for mental health among specific populations: adults with a history of exposure to traumatic experiences who reside in Egypt, Germany, and/or Italy. In addition to corroborating longstanding evidence on the association between exposure to trauma and PTSD symptoms, this study shows that there is a relationship between past exposure to trauma and fear of contracting COVID‐19, perceived stress, and different health loci of control at an early stage in the pandemic (June 2020). Our three hypotheses have been supported. By comparing the control group—participants without a history of traumatic exposure—to participants with differing histories of trauma exposure (i.e., childhood, adulthood, or both), this study highlights a number of inequalities on different ecological levels: event‐level, individual‐level, and community‐level. Furthermore, it responds to and calls for further research focusing on low‐ and middle‐income countries, including Egypt, where a relatively higher prevalence of traumatic exposure exists and yet literature on the subject is scarce.

The COVID‐19 pandemic is a new type of traumatic stress that has impacted our mental health negatively, partly because it has continued for a long time and partly because it involved multiple stressors: fear of present or future infection, anxiety about money, disruption of routine, social isolation and lockdown‐related stressors, and grieving the loss of loved ones. Our first hypothesis, that participants who were exposed to ACEs and other potentially traumatic events perceive more COVID‐19‐related stress than those who have been exposed to ACEs only, those who were exposed to other potentially traumatic events, and those who have not been exposed to either, was supported. In other words, individuals who have been exposed to both ACEs and potentially traumatic events were more likely to suffer from fear of contracting COVID‐19 and perceived stress than those who were only exposed to one or the other. Nevertheless, the latter group reported more fear of the pandemic and perceived stress than the control group. This finding lends support to the harmful impact that cumulative exposure to trauma can have on the individual.

Pointing to the high prevalence of trauma exposure in some low‐ and middle‐income countries, such as Egypt in the present study, this finding is important for two reasons. Noting the association between past traumatic exposure and health locus of control within the framework of the COVID‐19 pandemic is an important takeaway from this study. In line with the literature on health locus of control (e.g., Mellon et al., 2009), our second hypothesis, which states that participants who were exposed to at least one type of potentially traumatic event are more likely to have higher scores on powerful others health locus of control, was supported. Since locus of control contributes to shaping individuals' behaviors in relation to their health, with internal health locus of control being linked to healthier behaviors, our findings suggest that individuals who were exposed to ACEs and/or potentially traumatic events might believe that their health depends on others, and thus adopt a passive attitude towards it. Hence, in the context of the COVID‐19 outbreak, this might translate into a sense of helplessness over one's own health, and perhaps a stronger or deeper belief in the preventive and protective measures imposed by the governments and health institutions. On the one hand, such a sense of helplessness might not induce negative consequences, given the stronger sense of trust in the system. On the other hand, this helplessness can be compounded by a low sense of trust in the system. Therefore, depending on the culture and healthcare infrastructure, health loci of control may also have an important impact on the individual's mental health, in addition to their COVID‐19 related behaviors, which has additional repercussions on their own well‐being as well as that of others.

Our third hypothesis, which states that exposure to traumatic events and PTSD symptoms predict fear of contracting COVID‐19, perceived stress, and external health locus of control (both in terms of chance and powerful others) was supported. In line with previous findings (Yehuda & LeDoux, 2007), these results further suggest that having experienced different types of traumatic experiences increases the likelihood of fearing the pandemic, feeling stressed, and having different loci of control. Such associations are worsened by the presence of PTSD symptoms, which means that individuals who have been exposed to traumatic experiences but don't necessarily suffer from PTSD are at risk of developing other mental health conditions. Moreover, those who suffer from PTSD, in addition to having been exposed to traumatic experiences in the past, are at a higher risk of developing mental health conditions.

Finally, we conducted preliminary exploratory analysis to investigate the differences and similarities when it came to fear of contracting COVID‐19, perceived stress, and health locus of control across three countries with different cultural backgrounds and different exposure to COVID‐19. The findings highlight differences for all the outcome variables. Although it is important to interpret these results with caution given the unequal sample sizes recruited from the three countries, these findings are consistent with the recent literature on COVID‐19, which showed that respondents from the Middle East expressed more fear when compared to their European counterparts (Ali et al., 2021). The higher the collectivistic orientation of a society, the higher the perceived vulnerability to infectious diseases. This suggests that individuals who feel higher interdependence and sociability with others—especially those who emphasize the integrity of the in‐group, such as one's own family, ethnic group, country—may be more worried of getting infected by external forces, such as foreigners (out‐group), or something unknown, like a novel virus. They are more worried of infecting other members of the in‐group, due to close relationships (Germani et al., 2020). This kind of belief creates strong feelings of helplessness, which are associated with anxiety. This becomes even more obvious when society attaches great importance to the integration of the individual and rejects any kind of behavior that is different from the norm. However, the emphasis on transcendent powers (i.e., the prevalence of religion) may decrease the tendency of individuals to blame themselves for the illness. Furthermore, the unequal availability of healthcare services is another factor that may influence health locus of control across cultures. In societies where health care services are less available, there may be an increased trend toward self‐reliance, fostering internality, or, conversely, an enhanced perception of the role of luck, chance, and fate when it comes to one's health (Stein et al., 1984). Hence, cultural factors must be taken into account when trying to understand participants' health behavior and how they interact with public health measures, and these aspects must be acknowledged in future responses to pandemics.

## 7 LIMITATIONS OF THE PRESENT STUDY

This study derives value from collecting data from three different countries at an early stage of the outbreak of COVID‐19, focusing on exposure to traumatic events both in childhood and adulthood, and assessing different types of health loci of control. The study has two main limitations. First, the sample is not representative of the general populations in the aforementioned countries, considering participants were only recruited via online channels. Second, there exists an unequal gender representation among participants. Last, there is a sample imbalance across the three countries; participants recruited from Egypt resemble the largest sample.

## 8 IMPLICATIONS AND FUTURE RESEARCH

While our results seem straightforward, they have several important implications. First, monitoring mental health is critical during a pandemic, as generalized fear and stress‐related behaviors among the public could impede infection control. Furthermore, pandemic‐related measures and general uncertainty represent an unprecedented stressor, a potentially traumatic event that may significantly impact our health in the long run. Second, the public health response to the COVID‐19 pandemic should include intervention and prevention efforts to address individual mental health conditions, which has further repercussions on community psychology. Community‐level efforts, including health communication strategies, should prioritize individuals with a history of exposure to traumatic events. This can be done through a stepped‐care approach to mental health, which has been and continues to be strongly recommended by the World Health Organization. Third, once the pandemic is over, its negative socioeconomic consequences may have a detrimental effect on peoples' mental health, which requires healthcare systems and providers the world over to anticipate the increased demand for psychosocial support. This is a great opportunity to fight against stigma. Fourth, the findings loudly call for more research to be conducted in Egypt.

Further studies are needed to better understand factors that can mitigate against the negative consequences of a collective disaster like COVID‐19 on psychological well‐being. In particular, emotion regulation strategies and resilience could moderate the detrimental impact of previous exposure to ACEs and potentially traumatic events. They can play an important protective role to prevent the development of psychopathological symptoms. Furthermore, future studies should focus on community‐level intervention and prevention efforts to promote social connectedness and support individuals at risk of serious mental health conditions. Finally, future research on planning and response to post‐pandemic mental health demands should be based on prospective, randomized, controlled, peer‐reviewed data whenever possible. It is our hope that research will continue into the ongoing psychological impacts of COVID‐19, while addressing inequalities in both academic and applied research in certain regions of the world.

## 9 CONCLUSION

This study identified participants with a history of past exposure to traumatic events as being at risk of developing psychopathological symptoms, especially if they presently suffer from PTSD. This study is part of a longitudinal study, involving a follow‐up at 6 months to assess PTSD symptoms related to COVID‐19. The study aims at identifying the factors that increase or contrast the risk of developing posttraumatic symptoms. Moreover, the exploratory analysis of the cross‐cultural differences breeds new research questions that concern the impact of ACEs and potentially traumatic events in the context of a global pandemic.

## CONFLICT OF INTERESTS

The authors declare no conflict of interest.

### PEER REVIEW

The peer review history for this article is available at https://publons.com/publon/10.1002/jcop.22815

## Data Availability

The data that support the findings of this study are available from the corresponding author upon reasonable request.
